# Hyperhomocysteinemia: Underlying Links to Stroke and Hydrocephalus, with a Focus on Polyphenol-Based Therapeutic Approaches

**DOI:** 10.3390/nu17010040

**Published:** 2024-12-26

**Authors:** Carmen Ortiz-Salguero, Marina Romero-Bernal, Ángela González-Díaz, Elaheh Sobh Doush, Carmen del Río, Miriam Echevarría, Joan Montaner

**Affiliations:** 1 Instituto de Biomedicina de Sevilla, IBiS, Hospital Universitario Virgen del Rocío, CSIC, Universidad de Sevilla, 41013 Sevilla, Spain; cortiz-ibis@us.es (C.O.-S.); mromero-ibis@us.es (M.R.-B.); elasob@alum.us.es (E.S.D.);; 2 Instituto de Biomedicina de Sevilla, IBiS, Hospital Universitario Virgen Macarena, CSIC, Universidad de Sevilla, 41004 Sevilla, Spain; 3 Department of Neurology, Hospital Universitario Virgen Macarena, 41004 Sevilla, Spain

**Keywords:** small vessel, homocysteine, polyphenols, hydrocephalus, stroke

## Abstract

Hyperhomocysteinemia (HHcy), characterized by elevated homocysteine (HCys) levels, is associated with increased risks of neurovascular diseases such as stroke or hydrocephalus. HHcy promotes oxidative stress, neuroinflammation, and endothelial dysfunction, disrupting the blood–brain barrier and accelerating neurodegeneration. These processes highlight HCys as both a biomarker and a potential therapeutic target in vascular-related neurological disorders. Current research suggests that polyphenols, known for their antioxidant and anti-inflammatory properties, may reduce HCys levels and offer neuroprotection. Polyphenols have demonstrated effectiveness in modulating oxidative stress and inflammatory pathways triggered by HHcy. These compounds may also upregulate enzymatic functions involved in HCys metabolism, thus reducing neurotoxicity. Furthermore, polyphenol-rich diets, like the Mediterranean diet, have been linked to lower HCys levels and a reduced incidence of neurovascular disorders. This review provides an overview of HHcy’s role in neurovascular pathologies and examines the therapeutic potential of polyphenols in managing HCys levels and preventing HCys-induced neurovascular damage.

## 1. Introduction

Homocysteine (HCys) is a non-essential amino acid with a crucial role in cellular metabolism. Elevated HCys levels are a known risk factor for cardiovascular and neurovascular diseases as they promote oxidative stress, neuroinflammation, and endothelial dysfunction. High HCys concentrations have been associated with increased blood–brain barrier (BBB) permeability, heightened platelet aggregation, and the activation of proinflammatory pathways, all of which can accelerate neurodegeneration and vascular injury. These associations position HCys as a potential therapeutic target, especially in neurological conditions where vascular health is critical, such as stroke and hydrocephalus.

Dietary habits, particularly those rich in B-vitamins and polyphenols, play a critical role in regulating homocysteine metabolism, providing an opportunity for dietary strategies to mitigate its associated risks. The effects of B6, B9, and B12 on HCys levels in neurovascular conditions suggest that these vitamins are essential for HCys metabolism, underscoring how dietary interventions may help reduce elevated HCys plasma levels. Recent research also indicates that polyphenols may lower HCys concentrations due to their antioxidative and anti-inflammatory properties. This review aims to summarize current evidence on HCys’s role in disease development and to examine dietary strategies, particularly the use of polyphenols, as preventive measures against HCys-related neurovascular damage.

## 2. Hyperhomocysteinemia

The elevated blood level of HCys, a condition known as hyperhomocysteinemia, is a risk factor for different cardiovascular and neurodegenerative diseases [1,2]. HCys can be detected in both plasma and cerebrospinal fluid (CSF). While the measurement in CSF is not very common in routine clinical practice, it can be used in research or to evaluate specific neurological diseases [3]. A variety of techniques are available to measure HCys levels, but the most used ones are immunoassay methods or high-pressure liquid chromatography (HLPC) [4]. Immunoassays are favored for their speed, automation, and ease of use, despite limitations in accuracy and the potential for overestimation. In contrast, HPLC, while offering superior precision and reliability, is more labor-intensive, requires specialized equipment, and is less suitable for high-throughput analysis. Despite these challenges, HPLC remains the preferred method for accurate HCys measurement in clinical practice. [5]. The choice of measurement technique can influence the clinical interpretation of HCys levels, impacting patient management and the understanding of the role of HCys in various diseases.

The physiological HCys level is considered normal between 5 and 15 μmol/L, with an optimal upper limit of 10 μmol/L [6]. Hyperhomocysteinemia is diagnosed when blood levels exceed 16 μmol/L. It is classified as mild when levels range between 16 and 30 μmol/L, moderate when levels are between 30 and 100 μmol/L, and severe when values exceed 100 μmol/L [7]. In recent years, HCys has shifted from being considered solely as a risk factor to a key biomarker. HCys is a component cause in multifactorial diseases and may reflect an unhealthy lifestyle [8].

**Figure 1:**
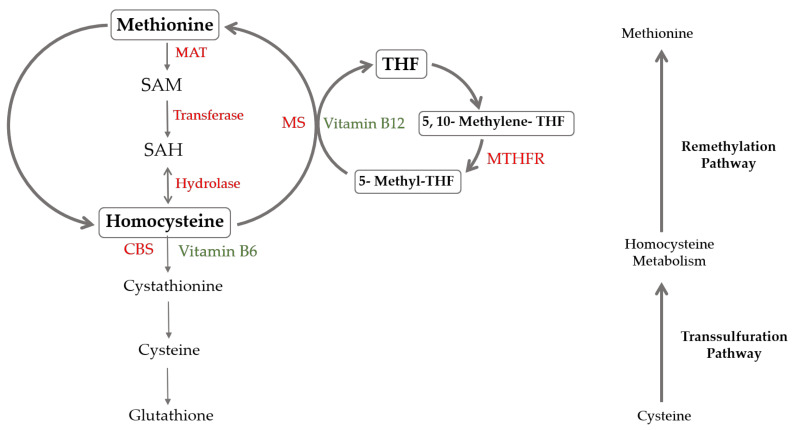
Schematic diagram of homocysteine metabolic pathways. Homocysteine is converted to methionine by MS enzyme, which utilizes vitamin B12 as a cofactor and accepts a methyl group from 5-methyl-THF, which is converted into THF. Then, methionine is converted to SAM through the activity of MAT. SAM is converted to SAH via a methyltransferase. SAH is finally hydrolyzed in homocysteine. Homocysteine is either remethylated back to methionine or transsulfurated to cystathionine by the vitamin B6-dependent enzyme, CBS. MTHFR is involved in the remethylation pathway, in which homocysteine is converted back to methionine. Abbreviations are as follows: CBS—cystathionine β-synthase; MAT—methionine adenosyl transferase; MS—methionine synthase; MTHFR—methylenetetrahydrofolate reductase; SAM—s-adenosyl-methionine; SAH—S-adenosylhomocysteine.

Multiple factors can contribute to hyperhomocysteinemia, including nutritional deficiencies, genetic alterations, and certain lifestyle habits. The most severe forms are typically associated with genetic mutations in the enzymes involved in the metabolism of HCys. The importance of these enzymes is their role at the branching point of two critical pathways in the methionine cycle: remethylation, regulated by 5,10-methylene tetrahydrofolate reductase (MTHFR); and transsulfuration, carried out by cystathionine-β-synthase (CBS) (Figure 1). The most prevalent genetic polymorphism worldwide is a mutation in the MTHFR gene, specifically a single nucleotide substitution (C to T) at position 677. This mutation produces a thermolabile enzyme variant with approximately half the normal activity, commonly associated with mild to moderate hyperhomocysteinemia. The prevalence of the 677C>T mutation varies by population; approximately 10–15% of Caucasians and Asians are homozygous for this mutation, while the heterozygous prevalence reaches 30–40% in some populations [9]. Another frequent mutation, the A1298C variant of MTHFR, also impacts HCys metabolism, though its effect on HCys levels is generally milder [10].

The most severe forms of hyperhomocysteinemia are associated with homozygous mutations in the CBS gene, which significantly elevate HCys levels while reducing cysteine synthesis. Although homozygous mutations in MTHFR and methionine synthase can also cause severe hyperhomocysteinemia, they are less common [11,12]. The prevalence of CBS deficiency-related homocystinuria is rare, estimated at 1 in 200,000 to 335,000 individuals worldwide [13].

On the other hand, hyperhomocysteinemia can also be due to deficiencies in the cofactors required for the metabolism of HCys, such as vitamins B6, B9, and B12. In the absence of these vitamins, HCys cannot be metabolized via either remethylation or transsulfuration and accumulates in plasma. Therefore, the deficiencies of these vitamins in the diet are significant contributors to hyperhomocysteinemia [14]. Dietary habits, particularly those rich in B-vitamins and polyphenols, play a critical role in regulating homocysteine metabolism, providing an opportunity for dietary strategies to mitigate its associated risks. Excessive alcohol consumption elevates hepatic S-adenosylmethionine levels, which in turn lead to an increase in HCys concentration. This association has been observed in patients with early-stage alcoholic liver disease and hyperhomocysteinemia [15]. Smoking has also been described to contribute to hyperhomocysteinemia by decreasing folate levels, thereby impairing the HCys remethylation pathway [16]. Other factors associated with increased plasma HCys include male sex, advanced age, and unhealthy diet. In contrast, a good physical condition and adherence to the Mediterranean diet, which is naturally abundant with these vitamins and antioxidant-rich foods, have been shown to lower HCys levels and reduce neurovascular risks [17].

## 3. Vascular Effects of Homocysteine

The role of HCys in contributing to endothelial dysfunction has been extensively documented. The endothelium consists of a single layer of endothelial cells that line the blood vessels, playing a crucial role in regulating vascular tone by controlling vasodilation and vasoconstriction. This regulation of blood vessel diameter is mainly mediated by nitric oxide (NO) [18]. In a typical physiological state, NO is generated in response to vasodilatory stimuli, such as shear stress, by endothelial nitric oxide synthase (eNOS) from L-arginin, requiring several cofactors like BH4y [19]. High levels of HCys have been shown to reduce BH4 availability in vitro, through both enhanced degradation and diminished synthesis [20]. Additionally, in vitro studies have shown that HCys leads to a reduction in the expression of the L-arginine transporter (CAT-1), limiting the substrate availability for NO production [21]. As a result of these processes, eNOS becomes uncoupled, leading to a reduced production of NO and ultimately disturbing the balance between vasodilation and vasoconstriction in the vascular system [22]. The uncoupling of eNOS contributes to heightened oxidative stress by producing reactive oxygen species (ROS) [23]. In fact, in vitro studies in endothelial cells have shown that the increase in ROS induced by HCys stimulates the secretion of proinflammatory cytokines like IL-6 and adhesion molecules such as ICAM-1 [24]. The proinflammatory environment generated by HCys leads to the activation of caspases 3 and 9, resulting in apoptosis-mediated cell death [25]. HCys is also implicated in other forms of cell death, such as pyroptosis [26] and ferroptosis [27]. In addition, a role for HCys in accelerating endothelial cell senescence has been reported [28].

The disruption of endothelial function caused by HCys accumulation is strongly linked to the progression of vascular diseases [29]. Research indicates that a hyperhomocysteinemia-inducing diet can greatly accelerate the development of atherosclerosis in vivo, which causes considerable plaque buildup in the arteries [30]. In addition, individuals with the homozygous form (C677T) of the MTHFR gene are more prone to hypertension [31]. Moreover, HCys has been highly correlated with increased prothrombotic activity in endothelial cells. HCys treatment leads to the externalization of phosphatidylserine, providing a procoagulant surface for platelet aggregation. Moreover, HCys promotes the formation of procoagulant microparticles that can enhance platelet activation and thrombin generation [32]. Research on a hyperhomocysteinemia rat model has also suggested a prothrombotic effect of HCys in vivo, observed through changes in blood composition, including decreased fibrinogen and increased platelet counts [33]. Moreover, this effect has also been confirmed for hyperhomocysteinemia on the activation of platelets in human plasma [33]. As part of the vascular system, HCys can also affect the BBB, a specialized network of blood vessels that tightly regulates what enters the brain. BBB dysfunction is a critical factor in the development of several conditions, such as AD [34], stroke [35], and hydrocephalus [36]. In addition to HCys’s known effects on the individual cells that form the BBB, such as endothelial cells and astrocytes, elevated HCys levels can disrupt the integrity of this barrier in vitro by reducing adherent proteins, which results in increased permeability [37].

In conclusion, elevated HCys levels contribute significantly to vascular dysfunction by impairing nitric oxide production, promoting oxidative stress, and inducing proinflammatory responses. Dietary patterns rich in processed foods and low in essential nutrients can exacerbate vascular damage induced by HCys [38]. These effects accelerate atherosclerosis, promote hypertension, and increase clotting risks, while also compromising the BBB’s integrity, which highlight a link between HCys and both cardiovascular and neurological diseases. Preventive strategies, including the adoption of balanced diets rich in antioxidants and B-vitamins, can mitigate endothelial dysfunction and reduce the risk of related vascular diseases [39].

## 4. Hyperhomocysteinemia and Stroke

Stroke is a neurovascular disorder produced via a significant reduction in the cerebral blood flow due to either an obstruction in the vasculature (ischemic stroke) or as a result of a bleeding caused by the rupture of brain vasculature (haemorrhagic stroke) [40]. Stroke represents the second most common cause of death and is a leading cause of disability globally [41,42]. Due to the reduced cerebral blood flow, there is a depletion of oxygen and glucose. This process leads to cell death within minutes in the ischemic core and the disruption of normal cell function in the surrounding area (penumbra) [43,44]. Cell death is followed by inflammation and ROS production, causing disruption in the BBB and brain edema [43,45].

The association between high levels of HCys and the risk of stroke has been extensively studied. Systematic reviews of observational studies have shown a strong, positive, and dose-related association between the serum concentration of total HCys and the risk of thrombosis and stroke, which is independent of other vascular risk factors [46,47,48]. In fact, it has been found that individuals with the MTHFR-TT genotype present higher levels of HCys and also have a greater risk of stroke than those with the CC/CT genotype [46,49,50].

Stroke is often associated with cerebral small vessel disease (CSVD), a group of clinical imaging syndromes often associated with stroke, underlying vascular cognitive impairment that is caused by cerebral vascular anomalies affecting small arteries, arterioles, capillaries, and venules [51,52]. CSVD is characterized by lacunar infarcts, white matter hyperintensities, cerebral microbleeds, enlarged perivascular spaces, and brain atrophy. It is considered an important cause of lacunar stroke and intracerebral hemorrhage [51]. Multiple studies have demonstrated that HCys levels are directly correlated with the presence of lacunar infarcts and inversely correlated with brain parenchymal fraction [52]. Hyperhomocysteinemia has also been reported to independently accelerate the progression of CSVD by elevating CSVD burden scores and, consequently, increase the risk of cognitive impairment [53]. Moreover, increases in HCys are associated with the development of different imaging markers of CSVD, such as cerebral microbleed, enlarged perivascular space, the presence of white matter hyperintensity, or lacunar infarction [53,54,55,56].

Although the specific mechanisms underlying the association between HCys and CSVD remain unclear, endothelial dysfunction is reported to play a crucial role. Hyperhomocysteinemia may compromise blood vessel architecture and induce endothelial dysfunction through different processes [48,57]. Recent evidence indicates that increased levels of total HCys are associated with histopathological features in vascular lesions (intimal thickening, elastic lamina rupture, smooth muscle hypertrophy, or platelet accumulation) through oxidative stress or inflammation processes, contributing to the association between serum HCys and CSVD [48,57]. Some factors such as smoking, renal impairment, atherogenic diet, cystine or folate deficiency, hypertension, diabetes, or hypercholesterolemia could also be contributing to the increased risk of stroke by affecting the HCys serum concentration [58,59]. This systematic review demonstrated that the strength of the association between homocysteine and risk of vascular disease varies according to study design. The association was strongest in cross-sectional and case–control studies, whereas prospective studies indicated less or, in some cases, no predictive ability for plasma homocysteine in cardiovascular disease [60]. Different hypotheses have proposed hyperhomocysteinemia as a general poor lifestyle biomarker [8]. Evidence has shown that a 5 µmol/L increase in HCys elevates the relative risk of ischemic stroke by 1.5 times, while a reduction of 3 µmol/L total HCys is associated with a 24% decrease in ischemic stroke [61]. Further studies are needed to establish HCys as an appropriate biomarker of stroke development and progression. Given the strong association between hyperhomocysteinemia and stroke, the modulation of the diet emerge as a promising preventive strategy due to the ability to lower homocysteine levels and counteract vascular damage.

## 5. Hyperhomocysteinemia and Hydrocephalus

Hydrocephalus is a condition characterized by CSF accumulation in the brain, resulting in elevated intraventricular pressure due to imbalances between CSF formation and absorption [62]. A disease commonly observed in older adults is the idiopathic normal pressure hydrocephalus (iNPH). It is characterized by a triad consisting of dementia, gait disturbance, and urinary incontinence, combined with ventriculomegaly, collectively referred to as Hakim’s triad [63]. The incidence of iNPH varies significantly by region. In hospital-based studies from the Netherlands, Germany, Sweden, Norway, and the USA, iNPH had an incidence of 0.5–1.2 patients per 100,000 inhabitants. In Japan, however, the incidence is notably higher, reaching 1.2/1000 inhabitants/year in people aged 70 years or older. A Norwegian study of iNPH in a defined population reported an incidence of at least 5.5 per 100,000 inhabitants/year, indicating that iNPH may be an underdiagnosed disease [64]. In iNPH, the origin of ventricles enlargement is still controversial, and mechanical, vascular, inflammatory, and metabolic factors have been indicated [65]. This ventriculomegaly exerts pressure on the surrounding structures and compresses periventricular arterioles and venules, which finally leads to poor tissue perfusion. The combined effects of hypoperfusion and mechanical compression trigger a number of pathophysiological changes in brain tissue, especially lesions in the periventricular white matter, but also in the gray matter. Other lesions include the following: alterations in metabolism, especially in glucose metabolism, which affect cellular energy balance; astrogliosis and neuroinflammation that worsen parenchymal stiffness and decrease brain distensibility; and alterations in the BBB, further complicating nutrient transport and waste clearance [66,67].

Another underlying mechanism of iNPH involves high concentrations of HCys in the CSF of patients. It has been suggested that high levels of this amino acid may reduce the distensibility of intracranial arteries, impacting the CSF dynamics. Typically, CSF diffuses into the subarachnoid space via pulsations of the intracranial arteries. Each arterial pulsation is accompanied by CSF absorption. As the distensibility of the arterial wall is reduced by the toxic effects of HCys, pulsations weaken, and CSF accumulates in the ventricles. Additionally, alterations of the BBB are also associated with elevated levels of HCys. Through mechanisms such as redox imbalance and oxidative stress, HCys leads to endothelial dysfunction and, ultimately, to the rupture of the BBB, which allows substances transported in the blood to enter the brain parenchyma. This imbalance between the vascular and ventricular system results in an abnormal osmotic gradient, driving water molecules into the ventricular system and leading to hydrocephalus [68,69,70].

The most common therapeutic approach to iNPH is the insertion of a shunt, which connects the cerebral ventricles to an alternative drainage site, usually the abdominal cavity. Lumbar drainage tests are used to decide whether shunt insertion is appropriate for a patient. To assess the patient’s progress after the shunt, biomarkers such as HCys can be used. Post-shunt, HCys levels in both plasma and CSF decrease progressively as excess CSF is removed, often improving gait, memory, and urinary symptoms as CSF HCys values normalize [71]. However, from CSF shunting until 2 years, plasma HCys levels may rise again, potentially re-triggering cognitive decline, reflecting neurodegenerative progression. Thus, plasma HCys monitoring serves as a valuable marker for tracking disease progression in shunt-treated patients [72]. For congenital hydrocephalus, although direct studies on HCys levels are limited, research suggests that genetic defects in this condition indirectly affect the folate pathway in which HCys is involved. Enzyme imbalances in folate metabolism observed in congenital hydrocephalus may predispose individuals to this condition, though HCys levels may not reach the elevated levels seen in iNPH due to differential folate utilization [73].

## 6. Homocysteine and Neurological Diseases

HCys is well known for its neurotoxicity, affecting both the viability of neurons and their ability to transmit signals. It affects the formation of functional neuronal networks, indicating that the effects of hyperhomocysteinemia are not only limited to the survival of neurons but also interfere with their proper functioning and connectivity [74]. Furthermore, HCys has been reported to impair hippocampal plasticity and synaptic transmission, resulting in learning and memory deficits, as observed in Alzheimer’s disease (AD) [75].

The mechanisms by which HCys contributes to neurodegeneration are multifactorial and complex and include excitotoxicity, oxidative stress, and mitochondrial dysfunction. HCys acts as an agonist of several glutamate receptors such as AMPA and NMDA. The overstimulation of these receptors causes an increase in intracellular calcium, inducing the production of ROS and the activation of caspases, finally leading to cell apoptosis [76]. HCys contributes to oxidative stress through multiple mechanisms that disrupt cellular redox balance and promote the generation of ROS. Elevated HCys levels inhibit key antioxidant enzymes [77]. HCys has been shown to interfere with the methionine cycle, reducing glutathione synthesis [78]. Furthermore, HCys upregulates NADPH oxidase, increasing superoxide production, and disrupts mitochondrial function, leading to further ROS accumulation [79].

HCys affects not only neuronal cells but also glial cells. Studies have shown that astrocytes exposed to elevated homocysteine levels exhibit cytoskeletal alterations, particularly in glial fibrillary acidic protein, leading to significant morphological changes. As a result of these structural alterations, astrocytes lose their ability to support essential functions, such as neurogenesis [80], prompting them to secrete proinflammatory factors, cytokines, and neurotoxic compounds that are damaging to the surrounding neurons [81]. In conclusion, hyperhomocysteinemia creates a hostile environment for CNS cells, promoting neurodegeneration and potentially contributing to BBB dysfunction. By modifying the integrity of the blood–brain barrier, HCys increases its permeability, which facilitates the infiltration of neurotoxic substances and immune cells into the brain parenchyma. This not only accelerates neuronal loss but also impairs the clearance of pathological proteins, such as beta-amyloid in Alzheimer’s disease, further amplifying disease progression [82].

Elevated levels of HCys have been considered a risk factor for developing neurological conditions such as AD or Parkinson’s disease [14]. Moreover, in AD, HCys levels are being investigated as a marker to assess disease progression. Higher and prolonged elevations in HCys are associated with increased neuronal damage, potentially accelerating the rate of cognitive decline [83]. In addition to its neurotoxic effect, HCys can also aggravate the symptoms that are already present in other neurodegenerative disorders. In pathologies such as Parkinson, where motor and behavioral symptoms predominate, elevated levels of HCys have been shown to worsen these deficits, exacerbating rigidity, tremors, and cognitive alterations [84].

HCyS has an important impact on vascular functionality, a factor increasingly recognized as a common denominator in various neurodegenerative diseases. A substantial body of evidence underscores the significant vascular component in conditions such as AD and Parkinson’s disease (PD). Notably, vascular alterations often accompany, and in some cases precede, the development of neurodegenerative pathology, suggesting a contributory role in the development of age-related dementia [85,86].

Levodopa, the most effective symptomatic treatment for PD, has been consistently linked to elevated HCys levels due to its metabolism via catechol-O-methyltransferase (COMT). Elevated HCys levels are associated with cognitive decline and structural brain changes in PD patients, such as increased ventricular volume as well as microscopic and macroscopic damage. Furthermore, research indicates that even before the initiation of levodopa therapy, PD patients with elevated HCys levels face a higher risk of cognitive impairment. Supplementation with vitamins B6, B12, and folate has demonstrated potential in countering levodopa-induced hyperhomocysteinemia and reducing its associated complications. However, despite these promising results, the integration of preventative supplementation into clinical practice remains limited, highlighting the need for further research and consensus on the therapeutic role of HCys management in PD [87].

Although there is ongoing debate about whether HCys functions as a biomarker or acts as a direct pathogenic agent, its involvement in driving the progression and development of neurovascular and neurodegenerative changes is evident. This warrants further investigation to inform on the development of novel preventive and therapeutic strategies.

## 7. Therapeutic Perspectives on Homocysteine Modulation

The metabolism of HCys is highly dependent on vitamin B6, vitamin B12, and folate; therefore, deficiencies in these nutrients can increase its serum concentration [88]. Elevated HCys is associated with a higher risk of vascular disease [89], as supported by evidence linking healthy diets, like the Mediterranean diet, with high dietary intake of vitamins and antioxidants, improper Hcy metabolism, and reduced cardiovascular risk [39,90]. Several studies have demonstrated that food fortification with folic acid, B6, or B12 vitamins is effective in reducing HCys levels [91,92,93,94,95], potentially reducing the prevalence of vascular diseases.

In the USA, a folate fortification policy introduced in 1998 led to reduced Hcy levels [96], and it was suggested to have contributed to the decline in stroke-related mortality [97]. Other studies have also suggested that folate and B vitamins supplementation may reduce the risk and improve prognosis of stroke by targeting elevated HCys levels. However, findings are mixed, as some studies report modest effects while others find no substantial impact on recurrent stroke or cardiovascular events. The Vitamin Intervention for Stroke Prevention trial assessed the effects of high-dose folic acid, B6, and B12 on patients with ischemic stroke, showing limited benefit in reducing recurrent stroke and myocardial infarction [98,99]. Additionally, a study in patients with type 2 diabetes highlighted that higher serum folate and B12 levels were associated with lower cardiovascular mortality, suggesting potential protective effects specific to populations with metabolic or vascular conditions [100]. Similarly, a systematic review and meta-analysis indicated that folic acid supplementation may be more effective in populations with low baseline folate levels, though results remain inconclusive across varied demographics and health backgrounds [101]. Dietary interventions with vitamins B6, B12, and folate have also been proposed to reduce the deleterious effects of HCys accumulation in hydrocephalus [102] and may potentially reduce the risk of congenital hydrocephalus [103]. However, the impact is modest, and further research is needed to clarify optimal intervention protocols. 

**Figure 2:**
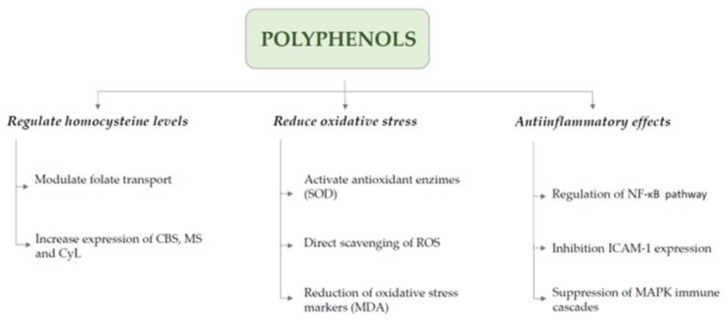
Role of polyphenols in hyperhomocysteinemia. Polyphenols mitigate HCyS-induced damage by regulating homocysteine levels and exerting antioxidant and anti-inflammatory effects. Abbreviations are as follows: CBS—cystathionine β-synthase; CyL—cystathionine γ-lyase; MDA—malondialdehyde; MS—methionine synthase; ROS—reactive oxygen species; SOD—superoxide dismutase.

Evidence has recently emerged showing that polyphenols may also reduce plasma HCys [104] and counteract the damage induced by high HCys levels [105] (Figure 2). In this sense, polyphenol-rich diets, such as the Mediterranean diet, are associated with a direct reduction in HCys but also with an increased risk of cardiovascular disease [106]. Polyphenols are secondary metabolites found in certain plants, known for their antioxidant, anti-inflammatory, and anti-apoptotic properties, as well as their antithrombotic effects [107]. Polyphenol-rich foods encompass a variety of fruits and vegetables, each characterized by distinct bioactive compounds. Some of the most prominent examples are apples (containing quercetin) [108,109], green tea (rich in catechins) [110,111], grapes and red wine (containing resveratrol) [112,113], turmeric (which has curcumin) [114], blueberries (rich in anthocyanins) [115], olive oil (containing hydroxytyrosol and tyrosol) [116,117], dark chocolate (high in flavanols) [118], spinach (with kaempferol and luteolin) [109], cinnamon (containing cinnamaldehyde and procyanidins) [119], and salicornia (rich in dicaffeoylquinic acids) [120].

Luteolin, a flavonoid polyphenol, has been shown to reduce homocysteine levels by promoting folate transport from the choroid plexus and increasing the activity of the proton-coupled folate transporter (PCFT). By maintaining an active folate pathway, luteolin helps prevent homocysteine accumulation, which is intricately linked to folate metabolism [121]. Other polyphenols, such as quercetin, work by increasing the mRNA expression of methionine synthase, cystathionine β-synthase, and cystathionine γ-lyase, enzymes involved in the remethylation and transmethylation of homocysteine, respectively, causing homocysteine to be reduced in the blood [122].

The antioxidant effects of polyphenols have been observed to work through the direct scavenging of ROS radicals [123] along with activating antioxidant systems such as the expression of antioxidant enzymes like superoxide dismutase (SOD) [124], thereby enhancing cellular resilience to oxidative stress. These effects are often accompanied by reductions in oxidative stress markers like malondialdehyde (MDA) levels [125]. Antioxidants, such as folate, and polyphenols, such as quercetin and resveratrol, have been shown to reduce hyperhomocysteinemia-induced oxidative stress by enhancing plasma antioxidant capacity, normalizing antioxidant enzyme levels, and increasing intraerythrocyte-reduced glutathione, a key molecule in oxidative stress defense [126,127]. HCys is also linked to lipid peroxidation and apoptosis. For example, resveratrol was able to decrease LDL oxidation, decrease DNA fragmentation, and reduce p53 mRNA expression, inhibiting HCys-induced cell apoptosis [128].

Polyphenols exert their anti-inflammatory effects through multiple mechanisms. For instance, quercetin promoted the anti-inflammatory M2 microglial phenotype, primarily through PI3K/Akt/NF-κB pathway regulation [129]. Similarly, resveratrol suppressed neuroinflammation by inhibiting NF-κB signaling, which reduces hippocampal inflammation and cognitive impairment in neuroinflammatory models [130]. Resveratrol suppressed immune activation cascades and mitogen-driven T-cell proliferation, thereby reducing HCys production and preventing its accumulation in the blood [131]. Similarly, olive oil phenolics inhibited HCys-induced ICAM-1 expression, a process associated with vascular damage. This effect was observed independent of the antioxidant activity of these phenolic compounds, suggesting that their protective role against HCys-induced endothelial dysfunction involves mechanisms beyond antioxidation [132]. Caffeic acid also demonstrated anti-inflammatory effects, particularly against hyperhomocysteinemia-induced inflammation in venular endothelial cells. This polyphenol inhibited HCys-induced leukocyte adhesion and rolling by reducing the expression of adhesion molecules, such as E-selectin and ICAM-1, in the cerebrovascular endothelium. Moreover, caffeic acid decreased ROS production and plasma levels of key chemokines involved in immune response and inflammation, including KC, MIP-2, and MCP-1 [133].

Other beneficial effects of polyphenols point directly to their action on kidney damage, with hypertension and hyperhomocysteinemia being the most common causes. Resveratrol reversed the effect of hyperhomocysteinemia on the kidney, increasing superoxide dismutase and glomerular filtration and decreasing the serum levels of MDA and urinary creatinine [134]. Recently, a polyphenol-rich extract was found to reduce HCys levels after three months of administration. This extract, which main polyphenols were quercetin, isorhamnetin, and dicaffeoylquinic acid derivatives, induced biochemical and proteomic changes related to cardiovascular health, such as lowered LDL, and increased GFR [135].

In addition, several polyphenols have been shown to modulate brain fluid dynamics by regulating the expression of aquaporins (AQPs). AQPs are a family of integral membrane proteins that function as selective channels to facilitate the transport of water molecules across cell membranes and are essential for brain fluid balance. The flavanone pinocembrin crosses the BBB, where it helps to mitigate brain edema, neuronal apoptosis, and endothelial cell damage by reducing inflammatory signals and decreasing the expression of AQP4. Curcumin has also been shown to dose-dependently reduce both gene expression and abundance of AQP4 and AQP9 proteins in a rat model of hypoxic–ischemic brain injury [136]. Epigallocatechin-3-gallate (EGCG) is also considered an anti-vasogenic edema agent that protects the integrity of the BBB. This compound regulates AQP4 expression in endothelial cells and astrocytes as well as inhibits the p38 MAPK-PI3K/AKT-eNOS signaling pathway in endothelial cells [137]. Similarly, tea polyphenols have also shown efficacy in reducing brain edema and protect the BBB through the regulation of the PI3K/Akt pathway and AQP4 levels. In particular, this type of polyphenol increased the levels of the anti-apoptotic protein Bcl-2 and tight junction proteins such us Zona Occludens 1 (ZO-1), claudin-5, and occludin, after subarachnoid hemorrhage [138,139].

These properties make polyphenols a promising therapeutic approach for conditions that are frequently associated with elevated plasma HCys levels. Stroke and hydrocephalus share risk factors such as aging and hyperhomocysteinemia, with overlapping symptoms including cognitive impairment and motor dysfunction. Both conditions involve disruptions in cerebral or cerebrospinal fluid flow, emphasizing the importance of early intervention for improved outcomes. 

Polyphenols have shown neuroprotective effects through multiple pathways in both hydrocephalus and ischemic stroke models, potentially due to their antioxidative and anti-inflammatory properties. In hydrocephalus-induced Wistar rats, a polyphenol extract from *C. sinensis* was administered intraperitoneally for 9 days and was found to reduce reactive astrocyte activity, as well as increase corpus callosum myelination, compared to untreated animals [140]. Similarly, treatment with EGCG, a polyphenolic compound found in green tea, has been shown to reduce periventricular oxidative damage in young rats with induced hydrocephalus through its capacity to eliminate ROS [141]. Additionally, studies have shown that curcumin can modulate the expression of AQP4, a protein critical for water transport in the brain and closely linked to the development of cerebral edema and hydrocephalus. By reducing AQP4 expression, curcumin helps limit cerebral edema, controlling water flow in the brain and decreasing fluid accumulation [142]. On the other hand, growing research highlights the preventive potential of dietary supplementation with polyphenols against neurovascular diseases, which have been extensively reviewed [102,120]. In fact, some of the beforementioned polyphenols have been shown to not only reduce the infarcted area in the brain in stroke models but also to improve cognitive functions by enhancing neurological score. For instance, quercetin reduced hippocampal neuron loss and brain edema as well as improved neurological function in rats when administered before the bilateral occlusion of the common carotid arteries [143]. In addition, vanillic acid [144] or kaempferol [145] demonstrated efficacy when administered as prophylactic treatments, while ferulic acid [146] or chlorogenic acid [147] showed benefits when administered after ischemia. These compounds share a common mechanism of action, which involves reducing inflammatory markers by neutralizing excessive free radicals and modulating inflammatory pathways such as NF-κB and TLR4, as well as inhibiting apoptosis. Moreover, a systematic review assessing the role of polyphenols on health outcomes in post-stroke patients highlighted that most of the studies found improvements in at least one of the study outcomes which included vascular function, blood pressure, or glycaemia [148]. Antioxidant supplementation with vitamin E, beta-carotene, and apple polyphenol extract over a 12-month period did not lead to significant improvements in functional recovery among stroke survivors. However, due to the controlled dietetic treatment, HCyS levels were reduced, highlighting the importance of monitoring the nutritional status in stroke patients [149]. Recently, a polyphenol-rich extract containing chlorogenic acid derivatives and flavonoid derivatives of luteolin, quercetin, and isorhamnetin was found to reduce HCyS levels and impact blood pressure in patients after transient ischemic attack, a well- established risk factor for future ischemic stokes [150]. However, studies with longer duration and larger sample sizes are needed to clarify how polyphenol-based therapies could mitigate vascular risk. Therefore, polyphenols, through their different mechanisms of action, are a promising therapeutic option in the treatment of neurovascular diseases. Their ability to influence multiple molecular pathways opens new possibilities for the development of effective treatments that can prevent or mitigate the effects of these diseases.

## 8. Conclusions

The reviewed evidence supports the therapeutic potential of lowering homocysteine levels in the prevention and management of neurovascular disorders. Elevated HCys levels correlate with increased vascular damage and neurological dysfunction, making it a promising biomarker and intervention target. While B-vitamin supplementation has shown some efficacy in reducing HCys, its effects on clinical outcomes for stroke and hydrocephalus are still under investigation. Polyphenols, known for their antioxidant, anti-inflammatory, and neuroprotective properties, offer an additional dietary approach to reduce HCys-related risks. More clinical studies are needed to establish optimal dosages, effective intervention protocols, and the long-term impacts of HCys-lowering strategies in different populations. Overall, integrating HCys-lowering interventions may contribute significantly to neuroprotection and improved outcomes in patients with stroke or hydrocephalus.
